# Anticancer and Antioxidant Activities of Rhizospheric Soil Bacteria of *Lophocereus marginatus*

**DOI:** 10.1155/ijm/1349429

**Published:** 2025-03-12

**Authors:** Jesica María Ramírez-Villalobos, César Iván Romo-Sáenz, Ramiro Quintanilla-Licea, Patricia Tamez-Guerra, Orquídea Pérez-González, José Roberto Estupiñan-Jiménez, Ángel David Torres-Hernández, Nancy Edith Rodríguez-Garza, Cristina Rodríguez-Padilla, Ricardo Gomez-Flores

**Affiliations:** ^1^Campus Cumbres, Universidad del Valle de México, Monterrey, Mexico; ^2^Departamento de Microbiología e Inmunología, Facultad de Ciencias Biológicas, Universidad Autónoma de Nuevo León, San Nicolás de los Garza, Mexico; ^3^Facultad de Medicina y Ciencias Biomédicas, Universidad Autónoma de Chihuahua, Chihuahua, Mexico; ^4^Departamento de Química, Facultad de Ciencias Biológicas, Universidad Autónoma de Nuevo León, San Nicolás de los Garza, Mexico; ^5^Departamento de Biología Celular y Genética, Facultad de Ciencias Biológicas, Universidad Autónoma de Nuevo León, San Nicolás de los Garza, Mexico

## Abstract

Identification of new sources of compounds with anticancer and antioxidant potential is essential for cancer treatment. Rhizospheric bacteria have emerged as a promising source of these agents. This study is aimed at investigating the anticancer and antioxidant properties of bacteria isolated from the rhizosphere of *Lophocereus marginatus*. Six rhizobacteria of *L. marginatus* were isolated and molecularly identified by sequencing the 16S rRNA gene and MALDI-TOF. We evaluated the inhibitory activity of methanol (MeOH) and ethyl acetate (EtOAc) extracts obtained from rhizobacteria against the murine lymphoma cell line L5178Y-R, as well as normal Vero monkey kidney cells and peripheral blood mononuclear cells (PBMCs). Compared with those of normal cells, the MeOH extract of *Paenibacillus* sp. PMS-B023 showed selective anticancer activity against lymphoma, with 50% inhibitory concentration (IC_50_) values of 117.5 *μ*g/mL and a selectivity index (SI) > 2.1, as compared with those of normal cells. L-Pro-L-Val cycle, gageostatin B, cinnamic acid, parvifloracin, daidzein, genistein, actinomycin D, actinomycin Y4, and surfactin C were detected in the MeOH extract, which may be responsible for its antilymphoma activity. We did not find significant antioxidant activity in any of the extracts evaluated using the 2,2-diphenyl-1-picrylhydrazyl technique (IC_50_ > 250*  μ*g/mL). The results showed that *Paenibacillus* sp. PMS-B023 has antitumor potential and selective activity against L5178Y-R lymphoma cells. The presence of anticancer compounds was also demonstrated, as previously described in the literature.

## 1. Introduction

Medicinal plants are commonly associated with microorganisms, possibly as a result of the production of unique secondary metabolites [1]. These interactions occur in different regions of plants, such as the phyllosphere (aerial part), endosphere (inner part), or rhizosphere (area of the soil influenced by roots) [2]. The rhizosphere is characterized by high microbial activity and diversity and is inhabited by fungi, algae, protozoa, and bacteria [3]. These microorganisms act as elicitors that may influence the production of bioactive compounds, such as alkaloids, steroids, and terpenes, by plants [4]. They also have the potential to produce metabolites that help plants survive environmental or biological factors [5].

Among the microorganisms found in the rhizosphere, bacteria produce metabolites of interest in medicine [6], such as compounds with antimicrobial [7], antioxidant [8], and anticancer [9] activities. However, scarce information on their potential to produce bioactive metabolites is available because the bacterial diversity is very large, and only a small fraction of the species are cultivable [10, 11]. Several studies have reported the biological activity of rhizobacteria that inhabit medicinal plants [12–14], including cacti [15]. However, this group of plants is still underexplored.

*Lophocereus marginatus* (Cactaceae) (synonym: *Pachycereus marginatus*) is a medicinal plant found in arid areas of Mexico [16]. Extracts of this species have been shown to exhibit antitumor activity in vitro and in vivo against murine lymphoma [17, 18]. It has been recently reported that endophytic fungi of *L. marginatus* produce compounds with antitumor activity in vitro against different types of cancer, such as breast, colon, and lymphoma [19]. However, the biological activity of the microorganisms that inhabit the rhizosphere of this species has not yet been investigated. The present study is aimed at determining the potential of the bacteria present in the rhizosphere of *L. marginatus* as producers of compounds with anticancer and antioxidant activities.

## 2. Materials and Methods

### 2.1. Isolation of Soil Bacteria Present in the *L. marginatus* Rhizosphere

Soil samples were collected from the rhizosphere of *L. marginatus* in San Nicolás de los Garza, Nuevo León, México, in May 2020. They were placed in sterile plastic bags and transported for processing to the laboratory. For bacterial isolation, we placed 1 g of soil in 9 mL of phosphate-buffered saline (PBS) at 70°C for 15 min, after which serial dilutions (10^–2^, 10^–3^, and 10^–4^) were prepared in PBS, and 100 *μ*L of the suspensions was plated by extension on potato and dextrose agar plates (PDAs; Difco Laboratories, Detroit, Michigan, United States) containing 3 g/L of yeast extract (Bio Basic, Canada) and 3 g/L of malt extract (Sigma–Aldrich Corporation, Oakville, Canada). The plates were incubated at 28°C ± 2°C for 3 weeks [20]. Colonies were then reseeded for purification on brain–heart infusion agar plates (BHI; TM Media, Titan Biotech LTD, Rajasthan, India) and preserved in BHI broth (TM Media) supplemented with 20% glycerol at −70°C until use.

### 2.2. Colonial and Cellular Morphological Characterization

Morphological characterization was visually determined by recording the shape, margin, elevation, and color of individual colonies that were plated on BHI agar. For cell morphology, Gram staining was performed, and cell morphology was observed at a magnification of 100× by optical microscopy (Carl Zeiss, Göttingen, Germany) [21].

### 2.3. Identification of Soil Bacteria by 16S rRNA Sequencing and Mass Spectrometry

Molecular identification of the isolated bacteria was performed by partial analysis of the 16S ribosomal RNA region. For this purpose, genomic DNA extraction from fresh cultures was performed using cetyl trimethyl ammonium bromide reagent (CTAB; Sigma–Aldrich, St. Louis, Missouri, United States), as previously reported [22]. The 16S rRNA region was amplified using universal primers for bacteria 27F (5⁣′-AGAGTTTGATCCTGGCTCAG-3⁣′) and 1392R (5⁣′-GGTTACCTTGTTACGACTT-3⁣′) [23] in a T100 thermal cycler (Bio-Rad, Singapore). PCR was performed in a volume of 50 *μ*L, using the Ruby Taq master mix 2X (Jena Bioscience, Jena, Germany). The PCR conditions consisted of an initial temperature of 95°C for 2 mins; 25 amplification cycles of 95°C for 30 s, 60°C for 30 s, and 72°C for 1 min; and a final extension of 72°C for 5 min. Amplified products were purified using an agarose gel extraction kit (Jena Bioscience). Product sequencing was performed with an ABI PRISM 310 TM Genetic (Applied Biosystems, Foster City, California, United States) sequencer at the Synthesis and Sequencing Unit of the Instituto de Biotecnología (IBT) at Universidad Nacional Autónoma de México in Cuernavaca, Morelos. The obtained sequences were assembled and edited using the BioEdit program (version 7.0.5.3) and then analyzed by the BLASTn tool of the National Center for Biotechnology (NCBI) (https://blast.ncbi.nlm.nih.gov/Blast.cgi) and the 16S-based ID database in EzBioCloud (https://ezibiocloud.net). DNA sequences were deposited at the NCBI, and accession numbers were obtained.

For identification by mass spectrometry, pure bacterial cultures were reactivated on BHI agar and analyzed by the direct colony method at MALDI-TOF (Microflex LT System, Bruker Daltonics, Bremen, Germany) using MALDI Biotyper 3.0 software in the Laboratorio de Infectología at Hospital Universitario “Dr. José Eleuterio González,” Universidad Autónoma de Nuevo León, México. The following criteria were used for the identification of isolates: A score of 2.0–3.0 was considered reliable at the species level, a score of 1.7–1.9 was considered reliable at the genus level but was considered unreliable at the species level, and a score < 1.7 was considered unreliable [24].

### 2.4. Fermentation and Preparation of Extracts

For the extraction of secondary metabolites, bacteria were activated on BHI agar plates for 24 h at 28°C ± 2°C, after which the bacterial suspensions were inoculated in 125 mL of BHI broth [25] and incubated at 28°C ± 2°C for 7 days at 150 rpm in an orbital shaker (MaxQ 400; Thermo Scientific, Waltham, Massachusetts, United States). After incubation, the cultures were centrifuged at 12,000 rpm (Sorvall ST16R centrifuge; Thermo Scientific, Pittsburgh, Pennsylvania, United States) for 15 min to separate the biomass from the supernatant [26]. Biomass was then macerated with MeOH at a 1:20 ratio, whereas the supernatant was extracted with EtOAc at a 1:1 ratio using a separation funnel. The extracts were then evaporated under reduced pressure in a rotary evaporator (Buchi R-3000; Brinkman Instruments, Inc., Westbury, New York, United States). Dry extracts were dissolved in dimethyl sulfoxide (DMSO; Sigma–Aldrich) at a final concentration of 25 mg/mL and stored at 4°C until use.

### 2.5. Cells and Culture Conditions

We used the murine lymphoma cell line L5178Y-R (ATCC CRL-1722), the normal control monkey kidney line Vero (ATCC CCL-81), and peripheral blood mononuclear cells (PBMCs), which were obtained from blood samples of healthy volunteers (40–50 mL), using the lymphocyte separation medium LymphoSep (Biowest, Nuaille, France) following the supplier's instructions. L5178Y-R cells and PBMCs were maintained in RPMI 1640 medium (Life Technologies, Carlsbad, California, United States), and Vero cells were maintained in modified Dulbecco's modified Eagle's medium (DMEM; Gibco, Life Technologies). The culture media were supplemented with 10% fetal bovine serum (FBS; Life Technologies) and 1% antibiotic–antifungal solution (Life Technologies). The cells were incubated in 5% CO_2_ in air (8000 DH Series; Thermo Scientific, Waltham, Massachusetts, United States) at 37°C in a humidified atmosphere [27].

### 2.6. Effect of Crude Extracts on Tumor and Normal Control Cell Growth

To determine cell viability, we used the colorimetric 3-(3,4-dimethylthiazole-2-yl)-2,5-diphenyltetrazolium (MTT; Invitrogen, Thermo Fisher Scientific, Sunnyvale, California, United States) reduction assay [28]. For this, L5178Y-R and Vero cells (1 × 10^4^ cells/well) or 1 ×10^5^ PBMCs were incubated in 96-well microplates (Corning Incorporated, Corning, New York, United States). After 24 h of incubation, the cells were incubated with 15.625 to 250 *μ*g/mL of the extracts for 48 h at 37°C in an atmosphere of 5% CO_2_ in air, after which 15 *μ*L/well of MTT was added at a final concentration of 0.5 mg/mL and incubated for 3 h at 37°C. We then added 150 *μ*L/well of DMSO to dissolve the formazan crystals and measured the optical density (OD) at 570 nm on a Multiskan GO microplate reader (Thermo Fisher Scientific, Rockford, Illinois, United States). We used 0.05 *μ*g/mL vincristine sulfate (Hospira, Warwickshire, United Kingdom) as the positive control and 1% DMSO as the negative control. The percentage of growth inhibition was determined by comparing the ODs of treated versus untreated cells. IC_50_ values were calculated using nonlinear regression, which was used to calculate the selectivity index (SI) by dividing the IC_50_ of the normal control cells by the IC_50_ of the L5178Y-R lymphoma cell line [29].

### 2.7. Antioxidant Activity

The antioxidant activity of the crude extracts was evaluated by the 2,2-diphenyl-1-picrylhydrazyl (DPPH) assay (Santa Cruz Biotechnology, Santa Cruz, California, United States) [30]. The 88 *μ*M DPPH solution was prepared in methanol, and 100 *μ*L was placed in 96-well microplates, after which 100 *μ*L of extract (15.6–250 *μ*g/mL) was added and homogenized. We used 250 *μ*g/mL ascorbic acid as a positive control (J.T. Baker, United Kingdom) and DMSO as a negative control. The plates were then incubated for 30 min in darkness, and the OD was read on a microplate reader (Thermo Fisher Scientific) at 517 nm on a Multiskan GO microplate reader (Thermo Fisher Scientific). The uptake of free radicals by the extracts was expressed as a percentage of the scavenging activity using the following equation [31, 32]:

 $$\begin{array}{c} \%\text{DPPH scavenging activity}=\frac{\;\left(\text{OD negative control}-\text{OD sample}\right)\;}{\text{OD negative control}}\times 100 \end{array}$$

The scavenging activity values were used to calculate the IC_50_ value through nonlinear regression.

### 2.8. Compound Identification by LC-MS^2^

The crude extract with the highest anticancer activity was selected for the identification of compounds by LC-MS^2^ analysis at the Laboratorio Especializado en Metabolómica y Proteómica of the Centro de Investigación Científica y Educación Superior de Ensenada (CICESE) located in Baja California, México, using an Agilent 1260 Infinity LC system (Agilent Technologies, Santa Clara, California, United States). Molecules were separated through a ProtID-Chip-43 II column (Agilent Technologies). The mobile phases consisted of H_2_O with 0.1% formic acid (FA) as solution A and acetonitrile with 0.1% FA as solution B. The column effluent was fed into a 6530 Accurate-Mass Q-TOF mass spectrometer (Agilent Technologies) to acquire compound mass data through an HPLC-Chip Cube MS interface, employing positive mode nanospray ionization.

### 2.9. Statistical Analysis

Statistical analysis was performed using GraphPad Prism 7 (GraphPad Software Inc., San Diego, California, United States). For the inhibition of growth in tumor and healthy cells, as well as antioxidant activity, the IC_50_ values and 95% confidence intervals were calculated using nonlinear regression. We used the D'Agostino–Pearson test to determine the normality of the data. The Kruskal–Wallis test was performed, followed by Dunn's test or one-way ANOVA followed by Tukey's test to determine differences between treatments with *p* ≤ 0.05. The results are expressed as the mean ± SEM of three replicates per treatment from three independent experiments.

## 3. Results

### 3.1. Isolation and Characterization of Soil Bacteria

**Figure 1:**
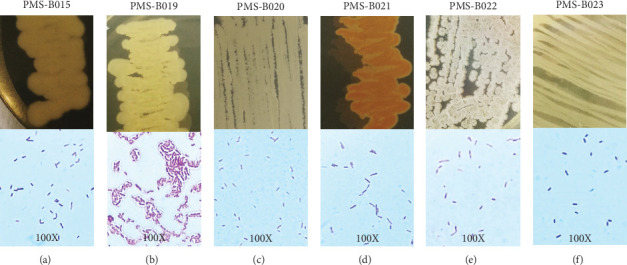
Rhizosphere soil bacteria from *L. marginatus*. (a) PMS-B015 strain. (b) PMS-B019 strain. (c) PMS-B020 strain. (d) PMS-B021 strain. (e) PMS-B022 strain. (f) PMS-B023 strain.

Soil bacteria associated with *L. marginatus* were isolated, and data from isolates that showed biological activity (PMS-B015, PMS-B019, PMS-B020, PMS-B021, PMS-B022, and PMS-B023 strains) are shown. The majority of isolates plated on BHI agar had colonies with irregular shapes and margins, domed elevations, and different shades. All the isolates were classified as Gram-positive, with *Bacillus*-shaped cells (Figure 1 and Table 1).

### 3.2. Molecular Identification and Mass Spectrometry of Soil Bacteria

We analyzed the 16S rRNA gene for the molecular identification of isolates. Only four of the six isolates were identified by comparing the sequences with the NCBI and EzBioCloud databases, showing a similarity of 96.6%–99.5% with species of the genera *Bacillus* and *Priestia*. Mass spectrometry was used to classify five of the six isolates with scores ranging from 1.8 to 2.4 as species of the genera *Bacillus* and *Paenibacillus*. The PMS-B022 isolate was not identified (Table 2).

### 3.3. Inhibition of Tumor and Normal Cell Growth

**Figure 2:**
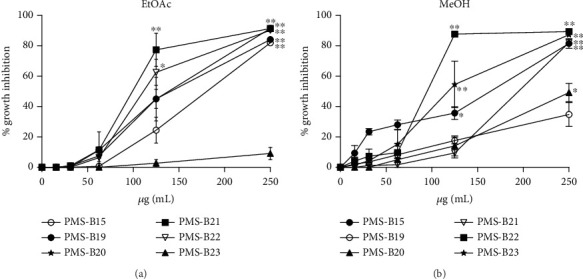
L5178Y-R lymphoma cell growth inhibition by soil bacterial extracts. (a) EtOAc extracts and (b) MeOH extracts. The data are presented as the means ± SEMs of three replicates from three independent experiments. ⁣^∗^*p* < 0.05 and ⁣^∗∗^*p* < 0.01, compared with the untreated control, using the Kruskal–Wallis test.

We evaluated L5178Y-R cell growth inhibition after 48 h of treatment with EtOAc and MeOH extracts from soil bacteria associated with *L. marginatus*. These compounds inhibited tumor cell growth in a concentration-dependent manner (Figure 2). The most active EtOAc extracts at 250 *μ*g/mL were *Bacillus* sp. PMS-B020 and *Bacillus amyloliquefaciens* PMS-B021, with 91.4% ± 0.01% (*p* < 0.01) growth inhibition (Figure 2a), whereas for the MeOH extracts, the PMS-B023 isolate presented the highest activity, with 87.3% ± 0.01% (*p* < 0.01) (Figure 2b). The IC_50_ values were determined for the L5178Y-R cells. Extracts that presented IC_50_ values lower than 250 *μ*g/mL were evaluated against normal PBMCs and Vero cells (Table 3). For tumor cells, the lowest IC_50_ values were obtained with PMS-B021, with 96.4 *μ*g/mL for the EtOAc extract and 90.3 *μ*g/mL for the MeOH extract with the PMS-B022 isolate. Regarding normal cells, the least toxic extracts were those of EtOAc with IC_50_ values > 250*  μ*g/mL for both normal cell types and the isolates PMS-B015 and PMS-B023 with IS > 1.9 and IS > 2.1, respectively. Vincristine sulfate at 0.05 *μ*g/mL (positive control) inhibited the growth of L5178Y-R cells, PBMCs, and Vero cells by 86.3%, 7.4%, and 3.8%, respectively.

### 3.4. Antioxidant Activity

The scavenging activity of EtOAc and MeOH extracts obtained from soil bacteria was determined to evaluate their antioxidant potential. At the highest concentration evaluated (250 *μ*g/mL), the EtOAc extract of PMS-B019 had the highest antioxidant activity (8.2%), and the MeOH extract of PMS-B15 had the highest activity (13.8%). All the extracts evaluated had IC_50_ values > 250*  μ*g/mL (Table 4). We used 250 *μ*g/mL ascorbic acid as a positive control, which showed 83.9% scavenging activity.

### 3.5. Compound Identification by LC-MS^2^

**Figure 3:**
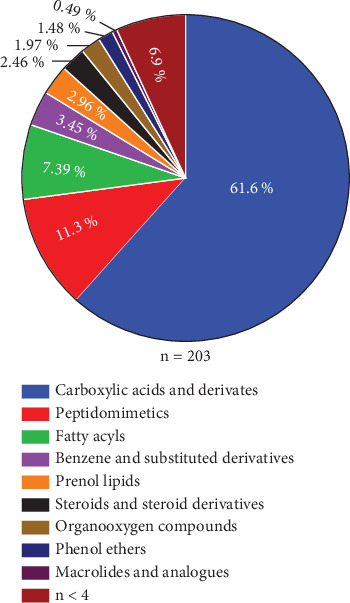
Percentage of chemical classes assigned to metabolites identified in the methanolic extract of *Paenibacillus* sp. PMS-B023.

**Figure 4:**
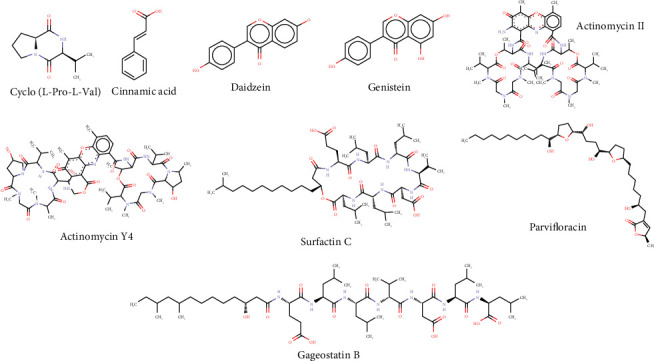
Putative structures of molecules with anticancer and antioxidant activity present in the methanolic extract of *Paenibacillus* sp. PMS-B023.

The methanolic extract of *Paenibacillus* sp. PMS-B023 was characterized by LC-MS^2^ because of its high selectivity against L5178Y-R cells. We detected 812 spikes in MS^2^, of which 203 were classified according to their chemical categories, using the ClassyFire tool (Figure 3). Carboxylic acids and derivatives were found to account for most of the extract composition (61.6%). In addition, 71 compounds were detected at the structural level (Supporting Information (available here)), nine of which have been previously reported to have anticancer activity (Table 5, Figure 4).

## 4. Discussion

This study demonstrated the potential of bacteria present in the rhizosphere of *L. marginatus* for the production of compounds with selective anticancer activity against murine L5178Y-R lymphoma cells, which is consistent with other studies showing the in vitro cytotoxicity of rhizobacteria from *Ibervillea sonorae* [20], *Taxus chinensis* [42], and *Clematis mandshurica* [43] against different cancer cell lines. The identified isolates corresponded to the genera *Bacillus*, *Priestia*, and *Paenibacillus*, which belong to the phylum Firmicutes, possibly because of the arid environment in which *L. marginatus* usually grows [16]. It has been reported that these genera are abundant in such environments due to their potential to resist drought [1].

*Bacillus* species are ubiquitous in soils and are among the largest producers of bioactive compounds with antibacterial, antifungal, antioxidant, and anticancer properties [44]. In the present study, *Bacillus* isolate extracts were cytotoxic against L5178Y-R lymphoma cells (IC_50_ = 96–189*  μ*g/mL) (Table 3), which agrees with previous reports on the anticancer activity of *Bacillus* species. Furthermore, *Bacillus cereus* has been reported to have cytotoxic activity against the HepG2 (IC_50_ = 147*  μ*g/mL), Hep2 (IC_50_ = 97.9*  μ*g/mL), MCF-7 (IC_50_ = 150*  μ*g/mL), and HeLa (IC_50_ = 300*  μ*g/mL) [44, 45] cell lines, and *B. amyloliquefaciens* species produce exopolysaccharides that inhibit the growth of the MCF-7 (IC_50_ = 70*  μ*g/mL), MC-4 (IC_50_ = 19.7*  μ*g/mL), and SGC-7901 (IC_50_ = 26.8*  μ*g/mL) [46, 47] cell lines.

*Priestia megaterium*, formerly known as *Bacillus megaterium* [48], is a large bacterium (2.5 *μ*m × 10*  μ*m) commonly found in plants and soils. It has significant biotechnological potential and has been used in various industrial applications [49]. In the present study, we showed that *P. megaterium* PMS-B019 possessed moderate activity (IC_50_ values of 100 to 500 *μ*g/mL [50]) against murine lymphoma cells (IC_50_ = 134.7*  μ*g/mL) (Table 3). This has been observed in other studies reporting the cytotoxicity of exopolysaccharides of *P. megaterium* against the HepG2 cell line (IC_50_ = 218*  μ*g/mL) [51] and in animal models as an adjuvant against fibrosarcoma [52] or as a chemosensitizing agent in breast cancer through the production of lipopeptides [53].

Bacteria of the genus *Paenibacillus* are usually found in soils and are often associated with plant roots [54]. They are of significant importance in agriculture, horticulture, industry, and medicine [55]. We found that the *Paenibacillus* sp. PMS-B023 methanol extract inhibited up to 87.3% of lymphoma cell growth at 250 *μ*g/mL (Figure 2). In contrast, other studies have shown the low cytotoxic activity of *Paenibacillus* strains. Exopolysaccharides from *Paenibacillus polymyxa* JB115 inhibited the growth of HeLa (33.5%), A549 (> 20%), Hep3B (> 20%), and sarcoma 180 (33.15%) cells at 250 *μ*g/mL [56], and the EJS-3 strain inhibited 55.37% BGC-823 cell growth at 400 *μ*g/mL [57].

In chemotherapy, drugs are expected to have high activity against cancer cells and minimal effects on healthy cells. Therefore, when assessing cytotoxicity in vitro, it is essential to calculate the SI, which determines the specificity of a drug or extract [58, 59]. The higher the SI is, the better the selectivity. SI values above 2 represent selective cytotoxicity [60]. We showed that only the methanol extract of the strain *Paenibacillus* sp. PMS-B023 presented SI values greater than 2 for the evaluated normal cells (SI > 2.1), making it a candidate for further studies. Further studies are necessary to determine whether the extract has any adverse effects on healthy cells. This includes conducting antihemolytic activity assays and in vivo evaluations to assess acute toxicity [61].

Various molecules and enzymes with antioxidant activity produced by microorganisms, including bacteria, have been reported [62]. These compounds donate electrons to free radicals to neutralize them, thus minimizing oxidative stress [63]. To consider an extract to have strong antioxidant activity, the IC_50_ values must be lower than 100 *μ*g/mL [64]. The isolates evaluated in this study did not show relevant antioxidant activity (IC_50_ > 250*  μ*g/mL). In contrast, other studies have demonstrated that strains of the same species or genera reported in this study presented high antioxidant activity, as measured by the DPPH test, with percentages of 67.3% for *B. amyloliquefaciens* VJ-1 (Kadaikunnan et al. 2015), 54% for *B. cereus* SZ1 [65], 94.7% for *B. megaterium* PFY-147 [66], and 45.4% for *P. polymyxa* EJS-3 [67], which may be due to the fermentation conditions used or the concentration of the metabolites present in the extracts responsible for the activity [68]. Other studies are consistent with our findings, indicating that the crude extract of the *Streptomyces* sp. BO-07 strain exhibited lower antioxidant activity (IC_50_ > 250*  μ*g/mL) compared to its anticancer activity against HepG2, HeLa, and Huh7 cell lines (IC_50_ 31–68 *μ*g/mL) [69].

LC-MS^2^ analysis of the methanol extract of *Paenibacillus* sp. PMS-B023 revealed metabolites previously described in the literature with anticancer, antimicrobial, antioxidant, and anti-inflammatory properties (Table 5), which, to our knowledge, have not been reported for the genus *Paenibacillus*. However, compounds found in this study have been identified in other species of bacteria. Gageostatins A–C have been extracted from *Bacillus subtilis*, where their anticancer activity has been demonstrated against six human cancer cell lines (breast cancer: MDA-MB-231, colon cancer: HCT15, prostate cancer: PC-3, lung cancer: NCI-H23, stomach cancer: NUGC-3, and kidney cancer: ACHN), with growth inhibition values (GI_50_) ranging from 12.2 to 23.2 *μ*g/mL [34]. In addition, actinomycins have been isolated from *Streptomyces* spp. [70], and surfactin C has been extracted from *B. subtilis* strains [71]. On the other hand, a study by Han et al. [72] showed that *Paenibacillus macerans* may glycosylate genistein, a compound with activity against ovarian cancer [73], prostate cancer [74], and colon cancer [75], which increases the activity of the compound. The production of compounds reported to have antioxidant activity (Table 5) has been demonstrated in bacterial species of the *Streptomyces* genus for cinnamic acid [76], daidzein, and genistein [77]. However, no significant antioxidant activity was observed in *Paenibacillus* sp. PMS-B023, which could be attributed to the concentration of these compounds not being sufficiently high in the crude extract. Of the 812 peaks detected in the analysis, only 71 compounds were identified at the structural level. This finding suggests the potential presence of novel compounds produced by *Paenibacillus* sp., which could hold promise for future biomedical applications.

## 5. Conclusions

Our results indicated that soil bacteria associated with *L. marginatus* have potential as producers of anticancer compounds for the treatment of lymphoma. The methanol extract of *Paenibacillus* sp. PMS-B023 showed the best selectivity against L5178Y-R cells and compounds with anticancer and antioxidant activities. Further studies are required to purify the secondary metabolites responsible for the activity in order to determine the mechanism of action underlying the anticancer activity.

## Figures and Tables

**Table 1:** Morphological characterization of the isolates.

**Isolate**	**Colony morphology**				**Cellular morphology**	
	**Shape**	**Margin**	**Elevation**	**Color** ^**a**^	**Shape**	**Gram stain**
PMS-B015	Irregular	Irregular	Flat	#EEDC82	Bacilli	+
PMS-B019	Circular	Even	Convex	#F0E68C	Bacilli	+
PMS-B020	Irregular	Irregular	Convex	#F1EDC2	Bacilli	+
PMS-B021	Irregular	Irregular	Convex	#CD853F	Bacilli	+
PMS-B022	Irregular	Even	Flat	#F6F9ED	Bacilli	+
PMS-B023	Circular	Even	Convex	#FAFAD2	Bacilli	+

^a^Colony colors were defined based on the web page http://www.webusable.com/coloursTable.htm (accessed in March 2023).

**Table 2:** 16S rRNA gene and mass spectrometry–based molecular identification.

**Isolate**	**16S rRNA gene**		**MALDI-TOF MS**		**Classification**	**Accession** ^**a**^
	**Closest relatives**	**% similarity**	**Identification**	**Score**		
PMS-B015	*Bacillus cereus* MW865710.1	96.6	*Bacillus cereus*	2.4	*Bacillus cereus*	*OQ174721*
PMS-B019	*Priestia megaterium* OL468358.1	99.5	*Bacillus megaterium*	1.94	*Priestia megaterium*	*OQ176214*
PMS-B020	*Bacillus velezensis* AY603658	99.24	*Bacillus subtilis*	1.8	*Bacillus* sp.	OQ176227
PMS-B021	*Bacillus amyloliquefaciens* MT579842.1	99.1	*Bacillus amyloliquefaciens*	2.2	*Bacillus amyloliquefaciens*	OQ176758
PMS-B022	ND	ND	ND	ND	ND	—
PMS-B023	ND	ND	*Paenibacillus* sp.	1.9	*Paenibacillus* sp.	—

Abbreviation: ND, not determined.

^a^Accession numbers of sequences were deposited with NCBI.

**Table 3:** IC_50_ (micrograms per milliliter) and SI of tumor and normal cells treated with EtOAc and MeOH extracts.

**Isolate**	**Extract**	**L5178Y-R**	**PBMCs**	**Vero**	**SI** ^**a**^
PMS-B015	EtOAc	168.2 ± 1.7 ab	134.6 ± 1.5 b	177.3 ± 1.8 b	0.8/1
	MeOH	127 ± 1.3 bc	> 250^b^	> 250^b^	> 1.9/> 1.9
PMS-B019	EtOAc	134.7 ± 1.8 ab	176.1 ± 1.6 bc	245.8 ± 1.1 b	1.3/1.8
	MeOH	> 250^b^	ND	ND	ND
PMS-B020	EtOAc	131.6 ± 1.5 ab	198.1 ± 1.8 c	> 250^b^	1.5/> 1.8
	MeOH	> 250^b^	ND	ND	ND
PMS-B021	EtOAc	96.48 ± 1.8 a	70.5 ± 1.7 a	211.5 ± 2 ab	0.7/2.1
	MeOH	189 ± 2 c	161.1 ± 1.6 bc	190.7 ± 1.4 ab	0.8/1
PMS-B022	EtOAc	112.1 ± 1.7 a	165.9 ± 1.5 bc	198.6 ± 1.8 ab	1.4/1.7
	MeOH	90.3 ± 1.6 a	83.4 ± 1.6 a	111.5 ± 1.9 a	0.9/1.2
PMS-B023	EtOAc	> 250^b^	ND	ND	ND
	MeOH	117.5 ± 1.3 a	> 250^b^	> 250^b^	> 2.1/> 2.1

*Note:* Values with different letters within the columns are significantly different (*p* ≤ 0.05) according to Tukey's test.

Abbreviation: ND, not determined.

^a^SI = IC_50_ of PBMCs or Vero/IC_50_ of L5178Y-R cells.

^b^IC_50_ values > 250*  μ*g/mL were not included in the statistical analysis.

**Table 4:** Scavenging activity of EtOAc and MeOH extracts.

**Isolate**	**EtOAc**		**MeOH**	
	**250 *μ*g/mL (%)**	**IC**_**50**_ **(*μ*g/mL)**	**250 *μ*g/mL (%)**	**IC**_**50**_ **(*μ*g/mL)**
PMS-B015	4.7 ± 2.1 abc	> 250	13.8 ± 1.4 e	> 250
PMS-B019	8.2 ± 1 cd	> 250	2.8 ± 0.7 ab	> 250
PMS-B020	3.6 ± 1.6 abc	> 250	5.2 ± 2 abc	> 250
PMS-B021	6.4 ± 1.2 bc	> 250	2.2 ± 0.7 ab	> 250
PMS-B022	0.9 ± 0.6 a	> 250	2.5 ± 1.8 ab	> 250
PMS-B023	0.9 ± 0.7 a	> 250	13 ± 3.4 de	> 250

*Note:* Values with different letters within the columns are significantly different (*p* ≤ 0.05) according to Tukey's test.

**Table 5:** Presumptive constituents of the methanolic extract of *Paenibacillus* sp. PMS-B023 reported anticancer activity.

**Compound**	**m**/**z**	**Molecular formula**	**Chemical class**	**Reported activity**	**Reference**
Cyclo(L-Pro-L-Val)	197.1	C_10_H_16_N_2_O_2_	Carboxylic acids and derivatives	Anticancer	[33]
Gageostatin B	1054.7	C_53_H_95_N_7_O_14_	Carboxylic acids and derivatives	Anticancer, antimicrobial	[34]
Cinnamic acid	149	C_9_H_8_O_2_	Cinnamic acids and derivatives	Anticancer, antioxidant, anti-inflammatory	[35]
Parvifloracin	611.5	C_35_H_62_O_8_	Fatty acyls	Anticancer	[36]
Daidzein	255	C15H10O4	Isoflavonoids	Anticancer, antioxidant, phytoestrogenic	[37, 38]
Genistein	271	C_15_H_10_O_5_	Isoflavonoids	Anticancer, antioxidant, phytoestrogenic	[37, 38]
Actinomycin D	602.3	C_58_H_82_N_12_O_16_	Peptidomimetics	Anticancer	[39]
Actinomycin Y4	645.3	C_61_H_84_N_12_O_19_	Peptidomimetics	Anticancer	[40]
Surfactin C	1036.7	C_53_H_93_N_7_O_13_	Peptidomimetics	Anticancer, antimicrobial	[41]

## Data Availability

The data that support the findings of this study are available from the corresponding author upon reasonable request.

## Supporting Information

Supporting Information Additional supporting information can be found online in the Supporting Information section. Metabolomic and chemoinformatic analyses of the methanolic extract of *Paenibacillus* sp. PMS-B023 using mass spectrometry.
